# Factors Affecting Specialty Training Preference Among UK Medical Students (FAST): Protocol for a National Cross-Sectional Survey

**DOI:** 10.2196/55155

**Published:** 2024-07-26

**Authors:** Tomas Ferreira, Alexander M Collins, Benjamin French, Amelia Fortescue, Arthur Handscomb, Ella Plumb, Emily Bolton, Oliver Feng

**Affiliations:** 1 Bristol Medical School University of Bristol Bristol United Kingdom; 2 Department of Clinical Neurosciences School of Clinical Medicine University of Cambridge Cambridge United Kingdom; 3 Department of Mathematical Sciences University of Bath Bath United Kingdom

**Keywords:** medical students, NHS, specialty preferences, workforce planning, medical education, National Health Service

## Abstract

**Background:**

The UK medical education system faces a complex landscape of specialty training choices and heightened competition. The Factors Affecting Specialty Training Preference Among UK Medical Students (FAST) study addresses the need to understand the factors influencing UK medical students’ specialty choices, against a backdrop of increasing challenges in health care workforce planning.

**Objective:**

The primary objectives of the FAST study are to explore UK medical students’ preferred specialties and the factors that influence these choices. Secondary objectives are to evaluate students’ confidence in securing their chosen specialty, to understand how demographic and academic backgrounds affect their decisions, and to examine how specialty preferences and confidence levels vary across different UK medical schools.

**Methods:**

A cross-sectional survey design will be used to collect data from UK medical students. The survey, comprising 17 questions, uses Likert scales, multiple-choice formats, and free-text entry to capture nuanced insights into specialty choice factors. The methodology, adapted from the Ascertaining the Career Intentions of UK Medical Students (AIMS) study, incorporates adjustments based on literature review, clinical staff feedback, and pilot group insights. This approach ensures comprehensive and nondirective questioning. Data analysis will include descriptive statistics to establish basic patterns, ANOVA for group comparisons, logistic regression for outcome modeling, and discrete choice models for specialty preference analysis.

**Results:**

The study was launched nationally on December 4, 2023. Data collection is anticipated to end on March 1, 2024, with data analysis beginning thereafter. The results are expected to be available later in 2024.

**Conclusions:**

The FAST study represents an important step in understanding the factors influencing UK medical students’ career pathways. By integrating diverse student perspectives across year groups and medical schools, this study seeks to provide critical insights into the dynamics of specialty, or residency, selection. The findings are anticipated to inform both policy and educational strategies, aiming to align training opportunities with the evolving needs and aspirations of the future medical workforce. Ultimately, the insights gained may guide initiatives to balance specialty distribution, improve career guidance, and improve overall student satisfaction within the National Health Service, contributing to a more stable and effective health care system.

**International Registered Report Identifier (IRRID):**

DERR1-10.2196/55155

## Introduction

In the United Kingdom, medical students typically undergo 5 years of undergraduate medical education, which may be extended if obtaining an additional degree during medical school, commonly known as an intercalated degree. Additionally, for those with previous degrees and pursuing a graduate-entry medical course, it generally spans 4 years. Following medical school, graduates enter the National Health Service’s (NHS) 2-year foundation program, exposing them to a variety of medical specialties. As specialty training commences immediately after the completion of the foundation program, those not intending to take a career break must apply to specialty training (also known as residency training) in the United Kingdom at the beginning of their second year of their career (Foundation Year 2). This underscores the early career decision-making required of medical graduates [1].

Postgraduate medical training in the United Kingdom follows 2 main pathways: “run-through” and “uncoupled” program. “Run-through” programs allow trainees to secure training posts from the outset and, subject to satisfactory progress, continue for the full duration of the program unencumbered by further application processes. Examples of run-through specialties include general practice, pediatrics, and ophthalmology. By contrast, “uncoupled” programs require trainees first to secure a training post at “core” level, in the broad specialty such as anesthetics, surgery, or internal medicine, and then again to enter “higher specialty training,” such as in cardiology or vascular surgery [2]. Notably, in the UK health care system, salary progression for doctors, whether in training or at the consultant level, is uniform across specialties. The primary factors that could influence the professional experience within the NHS are not solely the chosen specialty but include wildly variable aspects such as the duration of working hours, the intensity of the clinical environment, and the specific nature of clinical settings.

Since 2007, recruitment for much of specialty training in the United Kingdom has been centralized, using competitive national processes in which applicants are ranked according to various criteria [3]. Competition for specialty training in the United Kingdom is at an all-time high, having surged in recent years. In 2016, there were 20,044 applications for 10,671 posts aggregated across all specialties, conferring a ratio of 1.88 applications per post. By 2023, this ratio had risen to 3.37, with applications increasing by 249% while training places only grew by 183%. Some specialties, already renowned for being competitive have seen marked increases in their competition ratios. In neurosurgery, for example, the ratio climbed from 6.50 in 2016 to 12.75 in 2023. Meanwhile, even in specialties where the demand for services is exceedingly high—and rising—competition has intensified, with the ratio rising from 1.28 to 2.67 over the same period in general practice for instance [4]. Competition for residency training in the United Kingdom is set to worsen yet further, as the government announces plans to double medical school places by 2031/32 [5-8].

Medical students’ decisions may be influenced by their demographic backgrounds [9-13], experiences at medical school [14-16], and perceptions of the chosen specialty’s lifestyle, including work-life balance [17-19], financial incentives, and prestige [20]. External guidance from mentors [21] or family [22] and internal drivers, such as personal health experiences or the desire to serve particular patient demographics, also contribute to this complex decision-making process [22]. Although there have been international studies exploring which specialties resident doctors or medical students intend on pursuing and factors affecting these decisions [14,23-27], UK studies focused on why individuals are attracted to pursuing one specialty, and studies exploring which factors attract them to one specific specialty [28-44], there is currently a lack of broad, high-powered studies investigating the choice of specialty among UK medical students of all year groups and the factors influencing this preference.

Recently, we conducted the largest UK medical student study—the (Ascertaining the Career Intentions of UK Medical Students (AIMS) study—which investigated medical students career intentions and demonstrated that a noteworthy 35% of medical students intend to leave the NHS within 2 years of graduation [45,46]. Among our findings, we identified that a majority of medical students were dissatisfied with several aspects of residency training in the United Kingdom, including the cost of training, the rotational aspect of training, the ease of entry into a specialty training program, and the length and standard of training. This highlights the need to better understand medical students’ views, concerns, and expectations of specialty training in the United Kingdom.

We previously explored the role of medical schools in mitigating the current trends of medical students intending to leave the NHS. This work emphasized the importance of understanding and developing strategies within medical education to retain future doctors in the UK health care system. It also raised critical questions about the influence of medical school experiences, mentorship, and curricular design on career decisions [7,47].

The Factors Affecting Specialty Training Preference Among UK Medical Students (FAST) study seeks to understand the factors influencing specialty training choices among UK medical students. By examining these determinants across all student year groups, the study seeks to inform policy and educational strategies to better align training opportunities with the expectations and needs of the future medical workforce.

## Methods

### Study Objectives

#### Primary Objectives

The primary objectives of this study are (1) to examine the preference of UK medical students regarding their choice of specialty training and (2) to investigate the factors that influence UK medical students’ choice of specialty training.

#### Secondary Objectives

The secondary objectives of this study are (1) to assess the confidence levels among UK medical students regarding their ability to pursue their chosen specialty within the UK health care system; (2) to analyze the impact of demographic variables on students’ choice of specialty and their certainty and confidence in these choices; (3) to explore the association between academic variables and students’ specialty choice, as well as their certainty and confidence in these decisions; and (4) to identify and compare the variability in specialty choice trends and confidence levels across different UK medical schools.

### Study Design

FAST is a national, multicenter, cross-sectional study of UK medical students, following a similar methodology to the AIMS study [45]. Participants’ responses will be recorded via a web-based survey platform. The survey contains 17 questions. Questions are structured using a combination of Likert scale matrices, multiple-choice options, and free-text entry to broaden the capture of sentiment nuance and improve precision in the data. Data collection will take place from December 4, 2023, aiming to be completed on March 1, 2024. A full project timeline can be found in Table 1. This timeline will be approached with a level of flexibility to account for any national or center-specific challenges with data collection.

The survey consists of 3 sections. Section 1, to be completed by all participants, seeks background and demographic information. Consent is also obtained in this section. Section 2 gathers information on the certainty and choice of specialty as well as knowledge and guidance received regarding the pursuit of their chosen specialty. Section 3 invites participants to rate the importance of various factors influencing their specialty choice and provides an optional free-text entry for additional factors. The full questionnaire can be found in Multimedia Appendix 1.

To identify gaps in knowledge and inform the aim of the project, a review of the existing literature was conducted, including similar questionnaires and qualitative studies on students’ and doctors’ perspectives. Feedback from senior clinical staff was also obtained and used to revise questions based on the comments received. These steps were taken to ensure as nondirective and comprehensive a survey as possible, while remaining unencumbering so as to maximize the number of responses for statistical power. Further, an informal pilot group was convened, consisting of medical students from different stages of training. These students responded to the survey, and shared their experiences and perspectives on factors that influence their specialty training choices during a series of discussions. These informal discussions aimed to identify both positive and negative factors that could influence these decisions. The resultant feedback was critical in refining the survey’s contents to enhance participants’ experience.

The survey will be hosted on the Qualtrics survey platform (Provo, Utah, US), a General Data Protection Regulation–compliant web-based survey platform which supports both mobile and desktop devices. The survey will be distributed through multiple channels, including university mailing lists, student society social media pages, conferences, and personal and medical school social media platforms (such as Facebook, Instagram, LinkedIn, and X, formerly known as Twitter). To maximize distribution across the United Kingdom, a national network of approximately 200 FAST Collaborative members was recruited, representing all medical schools in the United Kingdom. Each member will be asked to liaise with their medical school dean to distribute the survey through official channels and advertise it at regular intervals over the collection period from December 2023 to March 2024.

**Table 1:** Project timeline.

Dates	Activity
October 1, 2023, to October 31, 2023	Conceptualization and planning phase
November 1, 2023, to December 3, 2023	Recruitment and training of factors affecting specialty training collaborative network across UK medical schools
December 4, 2023, to February 29, 2024	Data collection period
March 1, 2024, to August 1, 2024	Data analysis and manuscript preparation

### Study Population

All current students in all years at UK medical schools recognized by the General Medical Council and the Medical Schools Council are eligible to participate in the study. A list of eligible medical schools and approved programs can be found in Multimedia Appendix 2. At the time of writing, certain new schools, although approved by the General Medical Council, were yet to admit their first cohort of students, so they were excluded from this study.

### Data Collection

The survey will be disseminated largely through recruited medical student collaborators recruited prior to the study launch. These collaborators will ensure that their medical school is formally engaged at an early stage of this study and will be primarily responsible for disseminating this questionnaire among students at their medical school.

### Ethical Considerations

This study adheres to UK NHS Health Research Authority guidance, with NHS Research Ethics Committees’ review exemption applied. Participants will provide explicit consent prior to voluntarily completing the survey and are free to withdraw at any time by contacting the study team. All data analyzed are anonymous.

To obtain informed consent, a participant information sheet (Multimedia Appendix 3) will be made available to all participants. This information sheet will explain the rationale and purpose of the study, emphasizing its voluntary nature and the anonymity and confidentiality of participation. It will be made clear on the study materials and on the first page of the questionnaire that submitting responses provides consent for data usage. Additionally, the first question of the survey will obtain consent, and it will be mandatory to complete the survey. Consent can be withdrawn at any time, during or after completing the questionnaire by contacting the study lead. Email addresses will only be collected and stored for participants who provide explicit consent to this in the final question of the survey. Email addresses will be kept separate from the responses to survey questions, ensuring anonymity.

The anonymous responses will be collected and stored on the secure web-based server Qualtrics. Survey data will be extracted from Qualtrics and stored in a password-protected Microsoft Excel (Microsoft Corporation) file accessible only to the central study team. University of Cambridge standards for data handling will be followed for all data management and record keeping.

Participants will not receive compensation for completing the questionnaire. However, participants who provide their contact information will be entered into a prize draw, with one randomly selected individual receiving a cash prize of £250 (equivalent to US $316.42) at the end of data collection.

### Data Analysis

The forthcoming analysis of the FAST study data aims to construct a robust statistical representation of the factors influencing specialty training decisions among medical students. The initial step will involve descriptive statistics to establish a foundational understanding of the data set. This will include the calculation of frequencies, means, medians, and modes for the survey responses to provide a comprehensive picture of central tendencies and dispersion within the responses.

For the comparison of means among continuous variables across multiple groups, ANOVA will be used. This analysis will support discerning whether there are statistically significant differences in attitudes toward specialty training between different subgroups within the population, such as students from different academic years. In addition to these methods, logistic regression analysis will be performed to model the probability of certain outcomes, such as the likelihood of choosing a specific specialty based on a range of influencing factors. This will allow us to estimate odds ratios and understand the strength and direction of associations between multiple predictors and specialty choices. Moreover, discrete choice models will be used to analyze the choices made by medical students among a set of specialties. This method will provide insight into the relative preferences and the trade-offs that students make when considering various specialty attributes. Where appropriate, multivariate methods will be applied to simultaneously account for multiple influencing factors at once. This comprehensive approach will provide deeper insights into the complex interplay of variables affecting specialty decisions. Data analysis will be performed using Microsoft Excel (v16.71) and RStudio (version 4.2.1; Posit PBC).

Participants were given the opportunity to provide additional insights into factors influencing their specialty training decisions through free-text entries. These qualitative responses will undergo a thematic analysis to identify recurring themes. This qualitative approach will complement the quantitative analysis, providing nuanced insights into the factors influencing specialty training decisions beyond those captured by the survey questionnaire. By incorporating participants’ own perspectives and experiences, the thematic analysis will enrich the understanding of the complex interplay of variables shaping medical students’ career aspirations.

The study will adhere to the STROBE (Strengthening the Reporting of Observational Studies in Epidemiology) guidelines for cross-sectional studies [48].

## Results

Following study completion, it is intended that results will be presented at local, regional, national, and international conferences. In addition, the results will be disseminated via publication in a peer-reviewed medical journal. All collaborators will be given PubMed-citable collaborative coauthorship under the institutional name: *the FAST Collaborative*. We will have a hybrid authorship list of named authors and the institutional collaborative.

Following study completion, medical school collaborators will be able to request their institution-specific data and the analysis performed on said data from the FAST steering committee. These data will be anonymized. The fully anonymized data set will be made publicly available.

Data collection is currently underway, with the study being launched nationally on December 4, 2023. Data collection will close on March 1, 2024, and data analysis will commence. The results are expected to be available later in the year.

## Discussion

The FAST study anticipates significant variability in specialty preferences among medical students, influenced by the wide array of factors being explored in this investigation. Personal interests, prior experiences, and perceptions of the lifestyle conferred by pursuing different specialties are anticipated to be highly influential. We hypothesize that demographic factors, including gender, ethnicity, socioeconomic background, and geographical location, will also play a significant role in shaping specialty preferences. In addition, medical school experiences, such as teaching quality and clinical exposure, are expected to strongly influence students’ decisions regarding specialty training. The role of mentorship is predicted to impact specialty choice in a similar fashion. Other factors, including personal health experiences may also influence some students, while awareness of specialty training opportunities and current health care trends could shape students’ perceptions of certain specialties. Furthermore, we predict that knowledge of specialty pathways, application processes, and portfolio quality will increase as students progress through medical school, thus, leading to more informed decisions relating to specialization.

Although this study is centered on the UK health care system, the insights derived will have broader implications. The challenges and decision-making processes—and their associated challenges—faced by UK medical students are not unique to the NHS and often reflect wider global trends in medical education. For instance, factors influencing specialty choice, such as work-life balance and financial considerations, have been highlighted as also significant among medics in other health care systems [49-65]. This study’s findings can, therefore, offer valuable perspectives to international audiences, serving as a reference point for similar research conducted in other countries. Moreover, the detailed exploration of the UK system may help other nations identify and address similar challenges within their own medical education frameworks.

The FAST study is poised to provide a comprehensive examination of the factors influencing UK medical students’ preferred medical specialties. The strategic focus on a broad spectrum of elements, from work-life balance to mentorship, will yield critical insights valuable for educational institutions and policy makers in understanding the determinants of specialty selection. Furthermore, by incorporating a wide-ranging demographic profile, this study aims to discern patterns and potential inequities in the specialty choice process, affording a detailed perspective on how students’ different backgrounds might steer career trajectories within the medical field. Additionally, the study’s inclusive approach, encompassing students from all year groups and UK medical schools, will help to create a robust and diverse data set: one which offers a more accurate cross-section of the current student body and enhances the generalizability of the findings.

It is important to note the potential limitations of the FAST study. As with many cross-sectional studies, the FAST study’s main limitation is its snapshot view, which precludes tracking changes in students’ preferences and influencing factors over time. There is also the potential for selection bias, where students with certainty regarding their specialty training choices or strong opinions about factors influencing their decision-making may be more inclined to participate. Therefore, this could unintentionally exclude some students who are in the process of making these decisions. To address this, the study’s distribution strategy is extensive, involving a variety of channels and touchpoints to encourage a balanced response rate across the student population. The survey was also explicitly designed to be inclusive of those whose future specialty is uncertain. Recruitment and engagement challenges could also pose a risk to achieving a truly representative sample. Acknowledging this, extensive efforts are being made to secure a comprehensive and representative collaborator network to support student outreach. Finally, the survey design could restrict the depth of insights obtainable from predefined responses. However, the iterative process used during the extensive preparatory phase involving peer review and expert consultation, along with the provision for free-text responses, will ensure a rich and nuanced collection of data pertinent to specialty training choices.

The FAST study endeavors not only to inform but also stimulate strategic improvements in the way medical education is structured and how future doctors are supported with making decisions about their career. Insights gained could drive initiatives aimed at increasing the appeal of less popular specialties, improving mentorship programs, and refining the career guidance offered to medical students. These actions could potentially mitigate the specialty imbalances and contribute to a more even distribution of medical professionals across the health care system and increase retention rates of medical graduates in the NHS by providing a stronger sense of identity through specialty interest [7].

By capturing the preferences and perceptions of the future workforce, the FAST study will support the broader objective of establishing a regular, comprehensive survey of UK medical students, akin to a workforce census. This initiative could aid the government and educational authorities in their workforce planning strategies, ultimately fostering a more satisfied, fulfilled, and well-distributed medical workforce.

## Appendix

Multimedia Appendix 1 Copy of the Factors Affecting Specialty Training Preference Among UK Medical Students (FAST) questionnaire.

Multimedia Appendix 2 List of eligible medical schools and General Medical Council–approved programs.

Multimedia Appendix 3 Participant information sheet for the Factors Affecting Specialty Training Preference Among UK Medical Students (FAST) study.

## Abbreviations

AIMS: Ascertaining the Career Intentions of UK Medical Students
FAST: Factors Affecting Specialty Training Preference Among UK Medical Students
NHS: National Health Service
STROBE: Strengthening the Reporting of Observational Studies in Epidemiology

## Data Availability

The data sets generated and analyzed during this study will be made publicly available once the study is completed.
