# Supplementary material for: Factors Affecting Specialty Training Preference Among UK Medical Students (FAST): Protocol for a National Cross-Sectional Survey
Source: JMIR Res Protoc. 2024 Jul 26;13:e55155. doi: 10.2196/55155 (PMC11316162; doi:10.2196/55155)
Supplement: Multimedia Appendix 2 [file resprot_v13i1e55155_app2.docx]

**List of eligible medical schools and GMC approved programs**

Anglia Ruskin University School of Medicine

Aston University Medical School

Barts and The London School of Medicine and Dentistry

Brighton and Sussex Medical School

Brunel University London, Brunel Medical School

Cardiff University School of Medicine

Edge Hill University Medical School

Hull York Medical School

Imperial College London Faculty of Medicine

Keele University School of Medicine

Kent and Medway Medical School

King's College London GKT School of Medical Education

Lancaster University Medical School

Newcastle University School of Medical Education

Norwich Medical School

Plymouth University Peninsula Schools of Medicine and Dentistry

Queen's University Belfast School of Medicine

ScotGEM (A combination of St Andrew's and Dundee)

St George's, University of London

Swansea University Medical School

The University of Edinburgh Medical School

Ulster University, School of Medicine

University College London Medical School

University of Aberdeen School of Medicine and Dentistry

University of Birmingham College of Medical and Dental Sciences

University of Bristol Medical School

University of Buckingham Medical School

University of Cambridge School of Clinical Medicine

University of Central Lancashire School of Medicine

University of Dundee School of Medicine

University of Exeter Medical School

University of Glasgow School of Medicine

University of Leeds School of Medicine

University of Leicester Medical School

University of Liverpool School of Medicine

University of Manchester Medical School

University of Nottingham - Lincoln Medical School

University of Nottingham School of Medicine

University of Oxford Medical Sciences Division

University of Sheffield Medical School

University of Southampton School of Medicine

University of St Andrews School of Medicine

University of Sunderland School of Medicine

University of Warwick Medical School
