# Supplementary material for: Factors Affecting Specialty Training Preference Among UK Medical Students (FAST): Protocol for a National Cross-Sectional Survey
Source: JMIR Res Protoc. 2024 Jul 26;13:e55155. doi: 10.2196/55155 (PMC11316162; doi:10.2196/55155)
Supplement: Multimedia Appendix 3 [file resprot_v13i1e55155_app3.pdf]

FAST

University of BRISTOL | UNIVERSITY OF CAMBRIDGE

Participant Information Sheet

Factors Affecting Specialty Training choice among UK medical students: a national cross-sectional survey

Thank you for your interest in participating in this study. Please take a moment to read the following information. If you have any questions or concerns, please contact the principal researcher, Tomas Ferreira at tf385@cam.ac.uk.

What is the aim of this study? This study seeks to understand the factors influencing UK medical students' choice of specialty. We aim to identify key determinants in career decision-making and gather insights into the preferences and expectations surrounding specialty choices.

Why have I been selected to take part? All medical students currently studying at UK medical schools recognised by the General Medical Council (GMC) are being invited to take part in the questionnaire.

What do I have to do? If you decide to participate in this study, you will be asked to complete a questionnaire about your background, your specialty preferences, and the factors influencing these choices. This study is voluntary. If you choose to participate, you will be asked to complete the survey by clicking on the link found at the end of this document. The survey is expected to take about 4-6 minutes to complete, but there is no time limit. By submitting the survey, you consent to the collection and storage of data in accordance with the UK General Data Protection Regulation (GDPR) within the survey. For more information on GDPR, please click on the following link: https://gdpr-info.eu.

Do I have to participate? Participation is *entirely voluntary*. You may withdraw at any point during the questionnaire for any reason, before submitting your answers, by closing the browser. If you have already submitted data and wish to withdraw from the study, please contact tf385@cam.ac.uk by 29th of February 2024.

Who has approved this study? As per UK NHS Health Research Authority guidance, NHS Research Ethics Committees review exemption applied. All data are anonymous, and informed consent is obtained prior to participation.

How will my data be used? All answers will be anonymous, and we will take all reasonable precautions to ensure that they remain confidential. Data will be stored in a password-protected file and may be used in academic publications. Your IP address will not be stored. Prior to completion of data collection, we will store your institutional email address to confirm your student status. Research data will be stored for a minimum of ten years after publication or public release. General information about how the University uses personal data can be found here https://www.information-compliance.admin.cam.ac.uk/data-protection/research-participant-data

Who will have access to my data? Qualtrics is the data controller of the personal data held about you. Their privacy notice can be found here: https://www.qualtrics.com/privacy-statement. Qualtrics will share any email address you provide and your anonymised responses with the University of Cambridge, for the purposes of research as highlighted above.

Are there any benefits to taking part? While there are no immediate individual benefits, your participation will contribute to a greater understanding of the factors affecting specialty choices among medical students. This survey may also offer an opportunity for self-reflection on your future career plans. Additionally, all participants will be entered into a prize draw for a chance to win £250.

Will the research be published? The findings of this study may be published in peer-reviewed journals, presented at conferences, and a summary of the findings will be made available on social media.

Are there any possible risks involved with my participation? There are no anticipated disadvantages or risks associated with participating. If, however, you feel uncomfortable, you may stop the survey at any time.

Who do I contact if I have a concern or wish to complain? Please contact the principal researcher at tf385@cam.ac.uk.

How do I find out about the study's findings? The study is expected to conclude by March 2024.

Who to contact for further details? For any further questions or more information on the study, please email tf385@cam.ac.uk.

Link to the survey: https://cambridge.eu.qualtrics.com/jfe/form/SV_3rTuolxIM4Jjztc

Kind Regards,

Tomas Ferreira
FAST Study Lead
